# The Influence of Scar Patterns After Reduction Mammoplasty on Eye Movement and Gaze Pattern: An Eye-Tracking Investigation

**DOI:** 10.1007/s00266-023-03689-1

**Published:** 2023-10-18

**Authors:** Konstantin Frank, Rui Zeng, Stephanie Sedlbauer, Lukas Prantl, Riccardo Giunta, Sebastian Cotofana, Nicholas Moellhoff, Michael Alfertshofer, Kai Kaye, Vanessa Brébant

**Affiliations:** 1 Ocean Clinic, Marbella, Spain; 2 Department for Hand, Plastic and Aesthetic Surgery, Ludwig-Maximilians-University Munich, Munich, Germany; 3 Department of Plastic, Hand and Reconstructive Surgery, University Hospital Regensburg, Franz-Josef-Strauß-Allee 11, 93053 Regensburg, Germany; 4 Department of Dermatology, Erasmus Hospital, Rotterdam, The Netherlands; 5 Centre for Cutaneous Research, Blizard Institute, Queen Mary University of London, London, UK

**Keywords:** Scars, Reduction mammoplasty, Eye tracking, Gaze pattern, Breast surgery

## Abstract

**Background:**

Given that scars are acknowledged as the primary cause of postoperative dissatisfaction following reduction mammoplasty, it is imperative to comprehend the patient’s visual perception of different scar patterns in order to enhance patient satisfaction. To achieve this, eye-tracking technology provides an unbiased method of evaluating how observers assess breast scars.

**Methods:**

58 participants (32 females and 26 males) between the ages of 19 and 82 years (mean age of 29.47 ± 10.98 years) were shown 18 color photographs, taken at 3 viewing angles (right 45° oblique, frontal and frontal view with arms raised), from 6 patients undergone reduction mammoplasty with the inverted T-scar technique (3 patients) or no-vertical-scar technique (3 patients). The images were presented to every participant for a fixed duration of 5 s each. Eye-tracking device was used to collect and analyze the gaze data of viewers.

**Results:**

The nipple-areola complex (NAC) and the periareolar scar captured observers’ gaze faster, had longer duration and more count of eye fixation than all other parts of breast scars, regardless of the viewing angle and scar pattern. Moreover, the scar region in the inverted T-scar pattern received greater and faster visual attraction of observer’s gaze than the no-vertical-scar pattern.

**Conclusion:**

The NAC and the periareolar scar seem to be perceived as the most important regions for breast aesthetics. The findings can be helpful to assist plastic surgeons in determining the most appropriate technique for reduction mammoplasty, meanwhile underlining the importance of a fine periareolar scar and symmetric NAC for excellent aesthetic outcomes.

- This is to our best knowledge the first study using eye-tracking technology in evaluating reduction mammoplasty outcomes.
- This study explored the influence of different scar patterns after reduction mammoplasty on eye movements and gaze patterns among observers.
- The study have validated the significance of the NAC and the periareolar scar for breast aesthetics and revealed that the scar region in the inverted T-scar pattern may be judged less visually attractive than the no-vertical-scar pattern.

**Level of Evidence V:**

This journal requires that authors assign a level of evidence to each article. For a full description of these Evidence-Based Medicine ratings, please refer to the Table of Contents or the online Instructions to Authors www.springer.com/00266.

## Introduction

Breast hypertrophy is known to cause physical and psychological distress in women. Physical symptoms may manifest as limits in physical activity, chronic shoulder and back pain, shoulder grooving, intertrigo in the inframammary folds, and mastodynia. Meanwhile, psychological symptoms such as poor self-esteem, insecurity, lack of confidence, and difficulty choosing appropriate attire may appear [1, 2]. To address these symptoms, reduction mammoplasty is currently considered the most effective treatment [3].

Reduction mammoplasty, commonly referred to as breast reduction surgery, is a surgical intervention that aims to reduce the overall size of the breasts, preserve nipple-areola viability and achieve an aesthetically appealing contour [4], which blends essential aspects of both aesthetic and reconstructive breast surgery. It has gained popularity, with 507,363 procedures worldwide in 2021 and a 19.0% increase compared to 2020, according to the latest global survey from the International Society of Aesthetic Plastic Surgery (ISAPS) [5].

Although reduction mammoplasty can result in a significant improvement in physical and psychological well-being, aesthetic complaints, especially poor scars, become the crucial determinant in postoperative patient satisfaction [6–10]. Hence, eradicating or minimizing visible postoperative scars is a common goal of plastic surgeons.

The inverted T-scar technique is the classic approach to reduction mammoplasty of which the scars are composed of three separate parts: a periareolar scar, a vertical scar, and an inframammary (horizontal) scar [6]. The vertical part is located visible in the middle of both lower quadrants of the breast and is often responsible for patient postoperative dissatisfaction [10, 11]. The no-vertical-scar technique only has a periareolar and an inframammary scar and has therefore gained popularity [2, 11, 12], which however relies of course on the given preoperative measurements of the breast. Studies based on questionnaires or rating scales have been conducted to evaluate the aesthetic results for the two scar patterns, including scar quality and patient satisfaction [1, 6, 10]; however, there remains a paucity of objective assessment.

Eye-tracking technology is an objective tool to quantify and analyze viewers’ gaze via monitoring their eye movements and gaze patterns on an image [13]. In the present study, potential dissimilarities in eye movements and gaze patterns among observers, who were presented with images depicting two distinct postoperative scar patterns, were examined to provide an objective basis for a further understanding about the influence of scars on the viewing patterns of breasts.

## Methods

### Study sample

58 participants (55% females, *n* = 32; and 45% males, *n* = 26) between the ages of 19 and 82 years (mean age of 29.47 ± 10.98 years) were recruited to participate in this observational study. No specific inclusion criteria were defined. Participants who had a significant visual impairment that prevented them from evaluating the displayed images or who were unable to have binocular vision (e.g., due to the loss of one eye) were not considered eligible as per the exclusion criteria. All participants included were laypersons who had no background in breast surgery. The study was performed between February 2023 and March 2023. Participants were notified before joining the study that their eye movements would be monitored while they observed images, and they agreed in writing to allow their data and images to be used. Ethical approval was granted by the REDACTED (IRB protocol number REDACTED) and was carried out in compliance with regional laws (REDACTED) and good clinical practices.

### Eye-movement analysis

The eye movements of 58 participants were recorded using a Tobii Pro Nano binocular eye-tracker (Tobii Pro AB, Stockholm, Sweden) following previously established protocols [14, 15]. The eye-tracking device was situated at the bottom of a laptop monitor (Surface Laptop 3, Microsoft, Redmond, WA), which measured 15 inches and had a screen size of 340 mm × 244 mm. The eye tracker had a frequency of 60 Hz and captured eye movements up to 65 cm away, as well as lateral and cranial distances of 35 and 30 cm, respectively.

### Visual stimulus

**Fig. 1:**
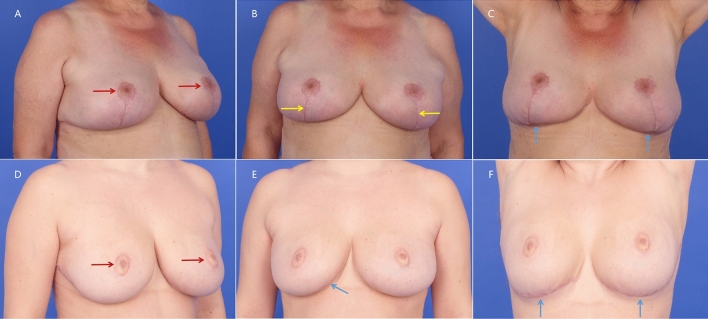
Postoperative images of patients undergone reduction mammoplasty in the inverted T-scar technique (above) or no-vertical-scar technique (below) at right 45°oblique view (**A** and **D**), frontal view (**B** and **E**), and frontal view with arms raised (**C** and **F**). Red arrow: periareolar scar; Yellow arrow: vertical scar; Blue arrow: inframammary (horizontal) scar

The viewing stimuli consisted of 18 color photographs of 6 patients who had undergone reduction mammoplasty with the inverted T-scar technique (3 patients) or no-vertical-scar technique (3 patients). All surgical procedures were performed at the plastic surgery department of REDACTED by the senior author (V.B.). Postoperative photographs of each patient were taken at various viewing angles, including right 45°oblique view, frontal view, as well as frontal view with arms raised (Fig. 1). The images were presented to the participants for a fixed duration of 5 s each.

### Data analysis

**Fig. 2:**
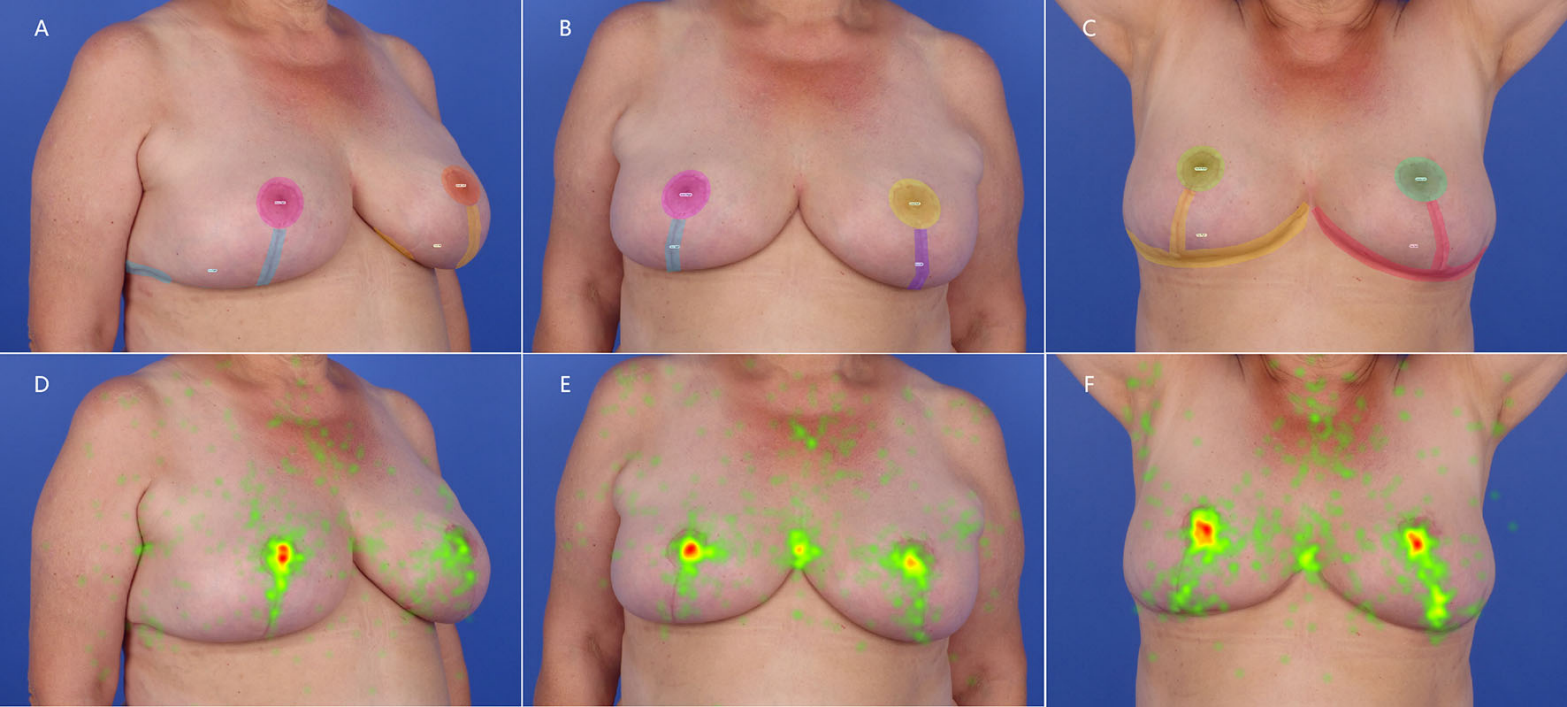
Images of a patient undergone reduction mammoplasty in the inverted T-scar technique, showing the breast AOIs (above) and heat maps (below) at right 45°oblique view (**A** and **D**), frontal view (**B** and **E**), and frontal view with arms raised (**C** and **F**). Ellipse: areola region, including NAC and periareolar scar; Polygon: scar region, including visible vertical scar and inframammary (horizontal) scar (**A**–**C**). Warmer colors such as red in heat map: areas received more fixations; cooler colors such as green: areas received fewer fixations (**D**–**F**)

**Fig. 3:**
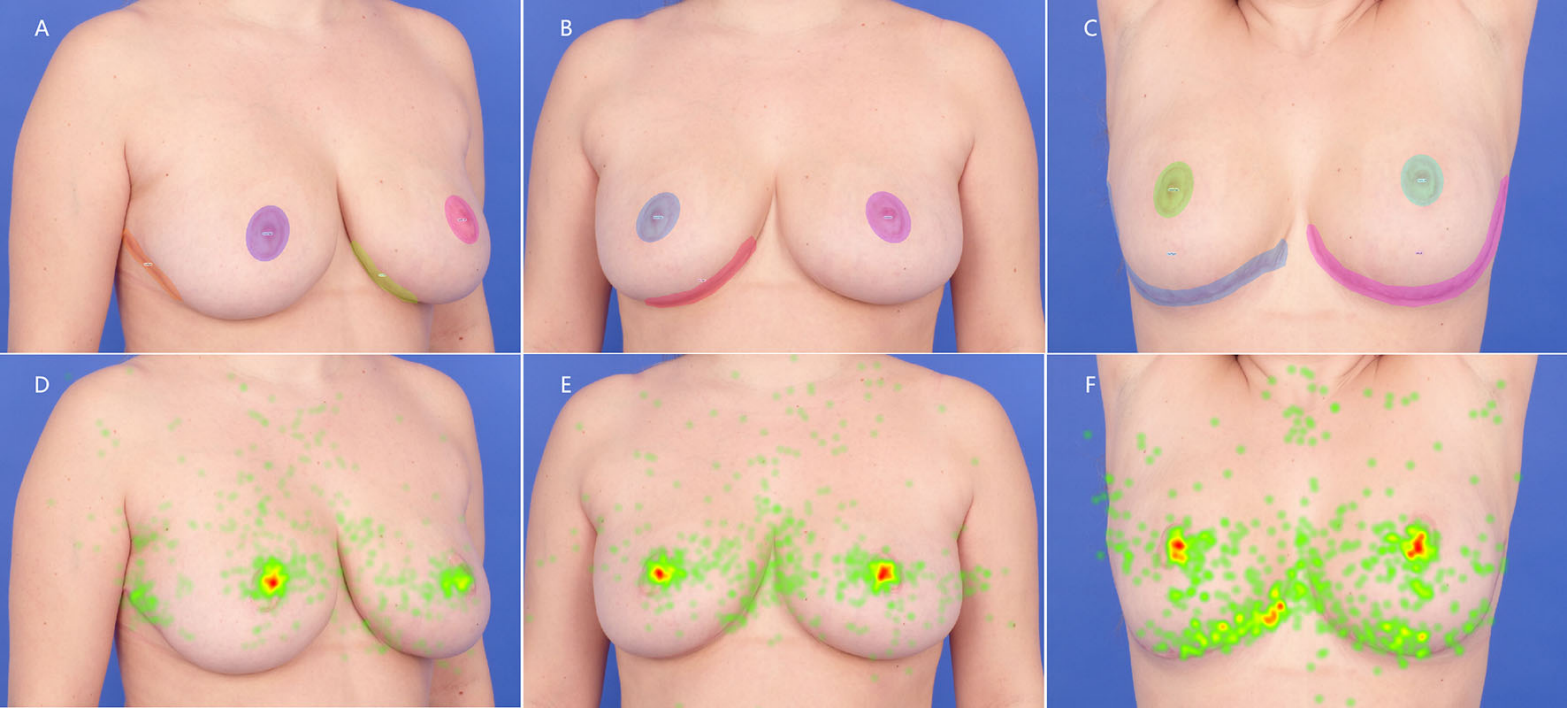
Images of a patient undergone reduction mammoplasty in the no-vertical-scar technique, showing the breast AOIs (above) and heat maps (below) at right 45°oblique view (**A** and **D**), frontal view (**B** and **E**), and frontal view with arms raised (**C** and **F**). Ellipse: areola region, including NAC and periareolar scar; Polygon: scar region, including visible inframammary (horizontal) scar (**A**–**C**). Warmer colors such as red in heat map: areas received more fixations; cooler colors such as green: areas received fewer fixations (**D**–**F**)

The captured data of eye movement pattern was processed with the eye-tracking internal software toolkit (Tobii Pro Lab). Areas of interest (AOIs) were defined in all images which included the nipple-areola complexes (NAC) and visible scars on bilateral breasts (scar region). If an area of scar was invisible in some viewing angle image, this area would not be marked as an AOI (Figs. 2A–C and 3A–C). The following variables were analyzed:

- Time until first fixation (interval between the onset of the visual stimulus and the initial eye fixation on the predetermined AOIs).
- Time of fixation (duration of eye fixation on the predetermined AOIs throughout the 5 s visual stimulus exposure).
- Number of fixation (amount of repeated eye fixations on the predetermined AOIs throughout the 5 s visual stimulus exposure).

### Statistical analysis

Tests for homogeneity of variance and normal distribution were performed which revealed nonnormally distributed (*p* < 0.001) data and unequal variance (*p* < 0.1). Differences in the variables of interest between the two scar patterns were therefore calculated using Wilcoxon rank sum test and among the different AOIs using Kruskal–Wallis test. To measure differences between participants, Wilcoxon rank sum test was performed to compare the responses between the two genders. SPSS Statistics 27 (IBM Corp., Armonk, NY) was used to carry out all the calculations, and a probability level of *p* ≤ 0.05 was used to determine the statistical significance of the results in order to draw appropriate conclusions.

## Results

### General results

No gender differences were observed in the eye movement pattern across the time of fixation and number of fixation (*p* > 0.05).

### Time of fixation

Regardless of the viewing angle and scar pattern, the areola regions always had a longer duration of a stable eye fixation throughout the 5-s stimulus exposure than the scar regions (*p* < 0.05). Only exception to this was the time of fixation between the left areola and right scar region at the right 45° oblique view of the inverted T-scar pattern image (*p* > 0.05) (Table 1, Figs. 2D–F and 3D–F).

**Table 1:** Mean time of fixation, time until first fixation and number of fixations for the respective AOIs of different visual stimulus (inverted T-scar pattern and no-vertical-scar pattern) values are mean ± standard deviation.

Variables	Time of fixation (s)			Time until first fixation (s)			Number of fixation		
	Inverted T-scar pattern	No-vertical-scar pattern	*p*	Inverted T-scar pattern	No-vertical-scar pattern	*p*	Inverted T-scar pattern	No-vertical-scar pattern	*p*
*Oblique*									
Areola right	1.71 ± 0.95	1.54 ± 0.95	0.087	0.41 ± 0.49	0.75 ± 0.83	< 0.001*	4.74 ± 2.34	3.81 ± 2.12	< 0.001*
Scar right	0.19 ± 0.42	0.07 ± 0.22	< 0.001*	1.82 ± 1.21	3.14 ± 1.32	< 0.001*	0.57 ± 1.16	0.21 ± 0.53	< 0.001*
Areola left	0.30 ± 0.44	0.25 ± 0.37	0.374	2.06 ± 1.05	2.18 ± 1.13	0.549	0.83 ± 1.11	0.74 ± 1.01	0.449
Scar left	0.08 ± 0.23	0.05 ± 0.16	0.125	2.52 ± 1.04	3.10 ± 1.11	0.098	0.22 ± 0.54	0.14 ± 0.42	0.119
*p*	< 0.001*	< 0.001*	–	< 0.001*	< 0.001*	–	< 0.001*	< 0.001*	–
*Frontal*									
Areola right	0.99 ± 0.69	0.73 ± 0.60	< 0.001*	0.93 ± 0.95	1.28 ± 1.15	0.002*	2.99 ± 1.75	1.91 ± 1.44	< 0.001*
Scar right	0.08 ± 0.19	0.06 ± 0.19	0.176	2.40 ± 1.39	2.15 ± 1.47	0.507	0.22 ± 0.44	0.22 ± 0.58	0.252
Areola left	0.74 ± 0.62	0.81 ± 0.77	0.739	1.19 ± 0.99	1.33 ± 1.20	0.691	2.35 ± 1.76	2.16 ± 1.68	0.326
Scar left	0.19 ± 0.37	0.02 ± 0.07	< 0.001*	2.03 ± 1.22	2.32 ± 1.78	0.580	0.66 ± 1.10	0.06 ± 0.23	< 0.001*
*p*	< 0.001*	< 0.001*	–	< 0.001*	0.005*	–	< 0.001*	< 0.001*	–
*Frontal with arms raised*									
Areola right	0.76 ± 0.67	0.47 ± 0.54	< 0.001*	1.30 ± 1.24	1.88 ± 1.39	< 0.001*	2.29 ± 1.61	1.29 ± 1.24	< 0.001*
Scar right	0.26 ± 0.39	0.21 ± 0.39	0.043*	1.62 ± 1.21	1.71 ± 1.14	0.545	0.82 ± 1.20	0.75 ± 1.33	0.097
Areola left	0.57 ± 0.53	0.55 ± 0.65	0.143	1.71 ± 1.36	1.64 ± 1.26	0.764	1.86 ± 1.52	1.47 ± 1.32	0.012*
Scar left	0.34 ± 0.42	0.17 ± 0.32	< 0.001*	1.49 ± 1.20	1.97 ± 1.30	0.015*	1.22 ± 1.42	0.61 ± 1.07	< 0.001*
*p*	< 0.001*	< 0.001*	–	0.015*	0.344	–	< 0.001*	< 0.001*	–

The vertical *p* value indicates the significance of the difference among the AOIs, and the horizontal *p* value indicates the significance of the difference between the scar patterns.

In comparing the mean time of fixation between the two scar pattern images, a significant difference was only identified in the right scar region at the right 45° oblique view (0.19 ± 0.42 s in the inverted T-scar pattern vs 0.07 ± 0.22 s in the no-vertical-scar pattern, *p* < 0.001). The right areola region (0.99 ± 0.69 s in the inverted T-scar pattern vs 0.73 ± 0.60 s in the no-vertical-scar pattern, *p* < 0.001) and left scar region (0.19 ± 0.37 s in the inverted T-scar pattern vs 0.02 ± 0.07 s in the no-vertical-scar pattern, *p* < 0.001) showed significant difference at the frontal view. At the frontal view with arms raised, there were statistically significant differences between two scar patterns images in the right areola region (0.76 ± 0.67 s in the inverted T-scar pattern vs 0.47 ± 0.54 s in the no-vertical-scar pattern, *p* < 0.001), the right scar region (0.26 ± 0.39 s in the inverted T-scar pattern vs 0.21 ± 0.39 s in the no-vertical-scar pattern, *p* = 0.043), and the left scar region (0.34 ± 0.42 s in the inverted T-scar pattern vs 0.17 ± 0.32 s in the no-vertical-scar pattern, *p* < 0.001). Although not all the comparisons of AOIs for time of fixation between two scar pattern images were statistically significant, longer time of fixation was still recorded for the scar regions in the inverted T-scar pattern than in the no-vertical-scar pattern (Table 1, Fig. 3).

### Time until first fixation

At the right 45°oblique view, the right areola region had a statistically significant shorter time until first stable eye fixation at 0.41 ± 0.49 s in the inverted T-scar pattern images and at 0.75 ± 0.83 s in the no-vertical-scar pattern images when compared to all other AOIs with *p* < 0.001. The time until first fixation for the other AOIs in the inverted T-scar pattern images was as follows: 1.82 ± 1.21 s for the right scar region, 2.06 ± 1.05 s for the left areola region, and 2.52 ± 1.04 s for the left scar region, with *p* < 0.001 across groups, while in the no-vertical-scar pattern images was as follows: 2.18 ± 1.13 s for the left areola region, 3.10 ± 1.11 s for the left scar region, and 3.14 ± 1.32 s for the right scar region, with *p* < 0.001 across groups.

At the frontal view of both scar pattern images, the areola regions showed a shorter time until the first stable fixation occurred compared to the scar regions (*p* < 0.05), despite not displaying significant differences between the right or left areola region and left scar region in the no-vertical-scar pattern images (*p* > 0.05).

At the frontal view with arms raised of both scar pattern images, no significant difference for time until first fixation was found between the different AOIs (*p* > 0.05), except a significant difference between the right and left areola region in the inverted T-scar pattern (*p* = 0.031) (Table 1).

By contrasting the mean time until first fixation between the two scar patterns images, at the right 45°oblique view, the significant differences were found in right areola region (0.41 ± 0.49 s in the inverted T-scar pattern vs 0.75 ± 0.83 s in the no-vertical-scar pattern, *p* < 0.001) and right scar region (1.82 ± 1.21 s in the inverted T-scar pattern vs 3.14 ± 1.32 in the no-vertical-scar pattern, *p* < 0.001). The right areola region revealed significant difference at the frontal view (0.93 ± 0.95 s in the inverted T-scar pattern vs 1.28 ± 1.15 s in the no-vertical-scar pattern, *p* = 0.002). At the frontal view with arms raised, time until first fixation in the right areola region (1.30 ± 1.24 s in the inverted T-scar pattern vs 1.88 ± 1.39 s in the no-vertical-scar pattern, *p* < 0.001) and the left scar region (1.49 ± 1.20 s in the inverted T-scar pattern vs 1.97 ± 1.30 s in the no-vertical-scar pattern, *p* = 0.015) were statistically significant between two scar patterns images. Despite no significant differences in other AOIs, shorter time until first fixation was recorded for almost all the scar regions in the inverted T-scar pattern than in the no-vertical-scar pattern (Table 1, Fig. 3).

### Number of fixation

The greater count of eye fixations during the 5-s stimulus exposure occurred in the areola region than the scar region, irrespective of the viewing angle and scar pattern (*p* < 0.001), except for the right 45° oblique view of the inverted T-scar pattern image where there was no significant difference between the left areola and right scar region (*p* > 0.05) (Table 1, Figs. 2 D–F and 3D–F).

When comparing the mean number of fixation between the two scar patterns images, at the right 45°oblique view, the significant differences were detected in right areola region (4.74 ± 2.34 in the inverted T-scar pattern vs 3.81 ± 2.12 in the no-vertical-scar pattern, p < 0.001) and the right scar region (0.57 ± 1.16 in the inverted T-scar pattern vs 0.21 ± 0.53 in the no-vertical-scar pattern, p < 0.001). The right areola region (2.99 ± 1.75 in the inverted T-scar pattern vs 1.91 ± 1.44 in the no-vertical-scar pattern, *p* < 0.001) and the left scar region (0.66 ± 1.10 in the inverted T-scar pattern vs 0.06 ± 0.23 in the no-vertical-scar pattern, *p* < 0.001) showed significant difference at the frontal view.

At the frontal view with arms raised, there were statistically significant differences between the two scar patterns in the right areola region (2.29 ± 1.61 in the inverted T-scar pattern vs 1.29 ± 1.24 in the no-vertical-scar pattern, *p* < 0.001), the left areola region (1.86 ± 1.52 in the inverted T-scar pattern vs 1.47 ± 1.32 in the no-vertical-scar pattern, *p* = 0.012) and the left scar region (1.22 ± 1.42 in the inverted T-scar pattern vs 0.61 ± 1.07 in the no-vertical-scar pattern, *p* < 0.001). Although number of fixations revealed no statistical difference in other AOIs, compared to images of the no-vertical-scar pattern, the images of the inverted T-scar pattern led to more count of eye fixations on scar regions, except the right scar region showing the same number of fixations in the two frontal scar pattern images (Table 1, Fig. 3).

## Discussion

The present study used eye-tracking technology to assess how the two distinct postoperative scar patterns of reduction mammoplasty influenced eye movements and gaze patterns of observers. Eye-tracking technology allows us to directly ascertain where a viewer is directing their gaze, rather than depending on self-reported preferences or ratings, by which the emotional response and involuntary perceptions to visual stimuli can be quantified [16–18]. This is to our best knowledge the first study using eye-tracking technology in evaluating reduction mammoplasty outcomes.

No gender differences were observed in the eye movement pattern across the time of fixation and number of fixations (*p* > 0.05), but a statistically significant difference in the time until first eye fixation could be measured between the two genders (*p* = 0.003). Previous study also showed gender difference of gaze patterns in some AOIs of female breasts [19].

Our results revealed that in three different viewing angles images for the two scar patterns, the areola regions always had a longer duration and greater count of stable eye fixations than the scar regions (*p* < 0.05), except between the left areola and right scar region at a right 45°oblique view of the inverted T-scar pattern image (*p* > 0.05). This may suggest that the focus of the observers was not mainly on the scar region, but the areola, regardless of the viewing angle and the scar pattern.

Several investigations have validated the significance of the NAC in the visual perception of the breasts. According to Pietruski et al, the NAC received the most attention when comparing women after unilateral mastectomy to healthy controls [18] and was also the primary focus in viewing a female torso, irrespective of the observer’s gender [19]. Cai et al emphasized the value of a reconstructed NAC in female breast reconstruction to distract from surgical scars [16]. Our previous research also demonstrated that gaze patterns between reconstructed and non-operated breast would aline only after reconstruction of the NAC [20]. The findings of the present study offer additional proof of the significance of NAC in breast aesthetics.

At the right 45°oblique view of both scar pattern images, the shortest time until the first fixation was recorded in the right areola region among all AOIs (*p* < 0.001), while at the frontal view of both scar pattern images, the areola regions showed the shorter time until the first stable fixation than the scar regions (*p* < 0.05), despite not displaying significant differences between the right or left areola region and left scar region in the no-vertical-scar pattern images (*p *> 0.05). These revealed that NAC represented the first areas of fixation by observers.

As already shown in previous studies, unharmonious features tend to garner an observer's gaze faster and the visual fixation on them persists for a longer duration [15, 21]. One could extrapolate that the NAC and the periareolar scar might be thought as less pleasing or attractive than the other parts of breast scars by observers. In the study conducted by Celebiler et al, patients who underwent T-scar reduction mammoplasty were most pleased with the periareolar scars and least pleased with the inframammary scars assessed by Likert scales [6]. The possible reason for the inconsistency in the findings of this study may be related to the differences in the origin of the observers and the methods of investigation. The observers in the study of Celebiler et al. were post-reduction mammoplasty patients, and the results might have been influenced by subjective factors of the patients due to the reliance on scale assessments. In contrast, the subjects of our study were non-surgical patients, and the study was based on subconscious and unbiased eye movements, uncovering the genuine preferences of viewers.

For the mean time of fixation between the two scar patterns images, although not all the comparisons of AOIs were statistically significant, longer time of fixation was still recorded for the scar regions in the inverted T-scar pattern than in the no-vertical-scar pattern, particularly in the frontal view with arms raised. A similar trend was found for the number of fixations, the images of the inverted T-scar pattern led to more count of eye fixations on scar regions, except the right scar region showing the same number of fixation in the two frontal scar pattern images. Notwithstanding significant differences only found among the comparisons of several AOIs, a tendency was discernible, wherein scar regions with the inverted T-scar pattern attained a quicker initial fixation time than those with the no-vertical-scar pattern. These outcome above indicated that the scar region in the inverted T-scar pattern received greater and faster visual appeal from the observer than in the no-vertical-scar pattern, from which it could be assumed that observers would perceive the scar region in the inverted T-scar as less aesthetically pleasing than in the no-vertical-scar pattern, according to previous investigations mentioned above [15, 21].

This aligned with the finding of a prior two-center study conducted by Hosnuter et al, which illustrated that using the no-vertical-scar technique can create the impression of an unsurgically altered breast by avoiding scars in the infraareolar area and making scars in other areas invisible. This leads to higher satisfaction for patients in the postoperative period [10]. However, as reported by Celebiler et al, patients who underwent T-scar reduction mammoplasty were least pleased with the inframammary (horizontal) scars assessed by Likert scales [6]. In the study carried out by Sprole et al. to evaluate patient preferences for breast reduction T-scar location via a designed survey, a considerable proportion of patients bothered by scarring expressed that the horizontal aspect was more troublesome, while the greatest number of respondents wished to remove the vertical scar if they had the option [8]. The possible explanation for this divergence might be that the inframammary scar caused the most discomfort by itchiness and irritation due to its proximity to the bra [8], while the vertical limb was deemed the most visually unacceptable due to its exposed position from frontal view, which was frequently reported as the primary source of postoperative patient discontent [8, 10, 11]. However, although the inframammary scar is generally located below the breast mound and not visible from the frontal view, when it extended beyond the concealed inframammary crease to the visible lateral chest wall, the axilla, or even the cleavage area, it might cause the complaint from the patients [6, 8, 11, 22].

According to the present study, eye-tracking technology provides a fresh perspective for the unbiased evaluation of a viewer's gaze in distinct postoperative scar patterns of reduction mammoplasty (including the inverted T-scar pattern and no-vertical-scar pattern), facilitating a deeper understanding of the breast reduction surgery features that attract the viewer's attention. The findings have validated the significance of the NAC in breast aesthetics and the crucial role the periareolar scar plays in this context, making it the “showcase” scar of breast reduction surgery [11], therefore producing an excellent periareolar scar should be an ideal goal for plastic surgeons performing this type of surgery. The circular feature of the areola may be compromised by the vertical scar with the inverted T-scar technique, whereas the no-vertical-scar technique offers the possibility of creating exceptional periareolar scars avoiding the inferior pulling force of a vertical scar contracture [10, 11]. Nevertheless, in most cases, avoiding the vertical scar will result in less favorable results regarding shape, upper pole fullness and longevity of the aesthetic result, so the benefits and disadvantages of each technique should be clearly discussed with the patient.

The results of this study indicate that it might be feasible to make prognostications about the personal aesthetic inclinations of a viewer using gaze data concerning different scar patterns after breast reduction surgery. This could be helpful to assist plastic surgeons in determining the most appropriate technique for reduction mammoplasty [23–26].

The study presents several noteworthy limitations. First and foremost, the study's exclusive focus on Caucasian subjects raises concerns about the generalizability of the findings to broader ethnic populations. Diversity in participants would have enhanced the external validity of the results and should be focus of future investigations. Another notable limitation pertains to the absence of subjective rating data concerning the perception of scars. The lack of information regarding participants' perceptions and comfort levels with the scars resulting from the two techniques diminishes the study's comprehensiveness and the inclusion of a separate vertical scar group would have been prudent. Moreover, the potential influence of breast volume reduction could have been assessed. Given that breast size could be an independent variable impacting gaze behavior, the study could have delved into investigating different levels of reduction as a potential parameter. By considering various degrees of reduction, the study could have provided insights into the potential relationship between breast size, scar length, and gaze patterns. Notwithstanding these limitations, it is important to acknowledge that the study's findings contribute to the existing body of knowledge surrounding breast reduction techniques and their visual impact. The utilization of eye-tracking technology, despite its limitations, represents a novel approach that sheds light on observer gaze patterns. The study's comparative analysis of two prominent techniques, even within the confines of its design, offers valuable insights that can guide future research endeavors.

## Conclusions

The present study evaluated if different scar patterns after reduction mammoplasty using either inverted T-scar technique or no-vertical-scar technique have an impact on eye movements and gaze patterns of observers by using objective eye-tracking technology. The NAC and the periareolar scar captured the observers’ gaze faster, and had a longer duration and higher count of eye fixations than the other parts of breast scars, regardless of the viewing angle and scar pattern. Moreover, the scar region in the inverted T-scar pattern had greater and faster visual attraction of observer’s gaze than the no-vertical-scar pattern. The findings have validated the significance of the NAC and the periareolar scar for breast aesthetics and revealed that the scar region in the inverted T-scar pattern may be judged less visually attractive than the no-vertical-scar pattern. The outcomes may contribute to assist plastic surgeons in better determining the most appropriate technique for their patient for reduction mammoplasty, meanwhile the results emphasized as well the necessity of generating an excellent periareolar scar.
